# Heterogeneities in *Leishmania infantum* Infection: Using Skin Parasite Burdens to Identify Highly Infectious Dogs

**DOI:** 10.1371/journal.pntd.0002583

**Published:** 2014-01-09

**Authors:** Orin Courtenay, Connor Carson, Leo Calvo-Bado, Lourdes M. Garcez, Rupert J. Quinnell

**Affiliations:** 1 School of Life Sciences, and Warwick Infectious Disease Epidemiology Research (WIDER), University of Warwick, Coventry, United Kingdom; 2 Laboratório de Epidemiologia e Imunologia aplicada às Leishmanioses, Seção de Parasitologia, Instituto Evandro Chagas, Belém, Pará, Brazil; 3 Centro do Ciências Biológicas e da Saúde, Universidade do Estado do Pará, Belém, Pará, Brazil; 4 School of Biology, University of Leeds, Leeds, United Kingdom; Institute of Tropical Medicine, Belgium

## Abstract

**Background:**

The relationships between heterogeneities in host infection and infectiousness (transmission to arthropod vectors) can provide important insights for disease management. Here, we quantify heterogeneities in *Leishmania infantum* parasite numbers in reservoir and non-reservoir host populations, and relate this to their infectiousness during natural infection. Tissue parasite number was evaluated as a potential surrogate marker of host transmission potential.

**Methods:**

Parasite numbers were measured by qPCR in bone marrow and ear skin biopsies of 82 dogs and 34 crab-eating foxes collected during a longitudinal study in Amazon Brazil, for which previous data was available on infectiousness (by xenodiagnosis) and severity of infection.

**Results:**

Parasite numbers were highly aggregated both between samples and between individuals. In dogs, total parasite abundance and relative numbers in ear skin compared to bone marrow increased with the duration and severity of infection. Infectiousness to the sandfly vector was associated with high parasite numbers; parasite number in skin was the best predictor of being infectious. Crab-eating foxes, which typically present asymptomatic infection and are non-infectious, had parasite numbers comparable to those of non-infectious dogs.

**Conclusions:**

Skin parasite number provides an indirect marker of infectiousness, and could allow targeted control particularly of highly infectious dogs.

## Author Summary

Zoonotic visceral leishmaniasis is a sandfly-borne disease of humans and dogs caused by the intracellular parasite *Leishmania infantum*. Dogs are the proven reservoir. The disease is usually fatal unless treated, and is of global health significance. Diagnosis of canine infections relies on serum antibody-based tests that measure infection. In some endemic regions, a test-and-slaughter policy of seropositive dogs forms part of the national control policy to reduce human infection. However, this strategy is not considered effective. Since not all infected dogs are infectious to sandfly vectors, one option is to target control at infectious dogs, as only these dogs maintain transmission. We quantify *Leishmania* numbers in individual host tissues from time of infection using molecular methods. Comparing these results with their infectiousness to sandflies, we also evaluate the performance of molecular and immunological assays to identify infectious animals. Parasite numbers varied substantially between individuals, increasing with duration and severity of disease. Infectiousness to the sandfly vector was associated with high parasite numbers, and parasite loads in the skin was the best predictor of being infectious. The results suggest that molecular quantitation is useful in identifying individuals and populations responsible for maintaining transmission, with potential application in operational control programmes.

## Introduction

Studies of microparasites usually consider hosts as homogeneous infection units (infected or uninfected), despite knowledge that infections progress through states of clinical severity, that clinical severity is often associated with the number of infecting microorganisms (load), and that individual transmission potential may be related to infection load. The significance of “super-spreaders” responsible for spreading infection to a disproportionate number of secondary cases has long been recognised [1], [2], however the relationships between parasite load and transmission are rarely measured; even in well-studied macroparasites (e.g. helminths) infectiousness is assumed to correspond to worm burden and egg count [3]–[6].

Variations in individual infection loads tend to be characterised by right-skewed (over-dispersed or aggregated) frequency distributions. Over-dispersion translates into diminishing proportions of the host population harbouring disproportionately higher infection loads. Where transmission potential is directly related to infection load, over-dispersed distributions may be interpreted as a small fraction of the population being responsible for most transmission, giving rise to the “20/80 rule” (whereby 20% of cases cause 80% of transmission), proposed for a number of parasitic agents (e.g. [7]–[10]). Heterogeneity in transmission can increase the basic case reproduction number *R_0_* of a pathogen compared to that under assumptions of homogeneous mixing or density-dependent contact networks [9], [11], and affect the effort required, and choice of strategy (mass or targeted), to interrupt transmission [7]–[9], [12]. Molecular techniques, such as real-time quantitative PCR (qPCR), have been used recently to differentiate between infected individuals and to help understand the spread and treatment of emerging infectious diseases e.g. [2], [9], [13]–[15], nevertheless few empirical studies relate individual infection loads to transmission.

Zoonotic visceral leishmaniasis (ZVL) is a fatal disease of humans and canids caused by the protozoan parasite *Leishmania infantum*, and transmitted between hosts by Phlebotomine sandflies. The domestic dog is the only proven reservoir [16], though severity of infection and infectiousness varies greatly between individuals; in humans and wild mammals the majority of infections are asymptomatic and non-infectious [16]. Control of ZVL focuses on the detection and elimination of infected dogs (particularly in South America), indoor residual spraying of insecticide, and human case treatment [17]. Positivity to serum anti-*Leishmania* antibodies is the principal criterion for mandatory slaughter of dogs [17]. Analyses indicate that this policy has little impact on reducing ZVL incidence, though robust data are lacking [16], and there have been calls to re-evaluate the ZVL control program in Brazil [16], [18]–[21]. Contributing factors to the lack of effectiveness include delays between testing and slaughter, low test sensitivity [22], and significant dog-owner non-compliance [21]. An alternative strategy could be to target infectious rather than infected dogs, providing infectious hosts can be identified. Direct measurement of infectiousness by xenodiagnosis requires blood-feeding of colony-reared sandflies on hosts followed by screening for parasite infections in the vector. Rearing large quantities of vectors for community surveillance however is not practical. Tissue parasite loads have the potential to provide a reliable indirect marker of infectiousness [23]–[30], though no studies have tested these relationships through the time course of infection.

Here we measure *L. infantum* loads in cohorts of naturally infected domestic dogs *Canis familiaris* and crab-eating foxes *Cerdocyon thous* in Amazon Brazil. This study is unique in being able to relate host tissue parasite loads to serial xenodiagnosis from time of natural infection. The aims were (i) to characterize the heterogeneities in *L. infantum* loads between sampled tissues and between individual hosts with different severity of infection, (ii) to investigate whether tissue parasite loads can predict infectiousness to the sandfly vector; (iii) to compare parasite loads between dogs and crab-eating foxes, and (iv) to evaluate the performance of qPCR and ELISA diagnostic assays to identify infectious animals in mixed populations.

## Materials and Methods

### Ethics statement

Canine samples were collected with informed consent from dog owners. Sampling was performed in accordance with UK Home Office guidelines.

### Study site and study design

Dog samples were available from −80°C archived material generated in a cohort study of naturally exposed dogs between April 1993 and July 1995 in the municipality of Salvaterra, Marajó Island, Pará State, Brazil, in which bone marrows aspirated from the iliac crest and 3 mm skin biopsy punches of the ear pinnae outer edge were sampled repeatedly at approximately 2 month intervals for up to 27 months post initial exposure [31]. Ear skin was the preferred skin sample since it is reported to be more infectious to sandflies than abdomen skin [23], [30]. Both skin and bone marrow are reported to be more sensitive than blood for parasitological and molecular detection of *L. infantum*, and higher qPCR counts are recorded in bone marrow than in blood [32]–[34]. For the present study, 265 bone marrow samples were available from 82 infected dogs (1–10 samples per dog), and 185 ear skin biopsy samples were available from 64 infected dogs (1–6 samples per dog), of which 173 samples from 63 dogs had paired bone marrow samples. Fox samples were collected during a concurrent longitudinal study of sympatric marked-recaptured free-ranging foxes [35]. Here, 67 bone marrow samples from 34 infected foxes, and 51 ear biopsy samples from 30 infected foxes, were available; all ear biopsy samples had paired bone marrow samples. Dog samples were collected with informed consent from dog owners.

### Assays

Dog and fox samples were assayed at all, or at the majority, of time-points, for (i) anti-*Leishmania* IgG by ELISA using crude leishmanial antigen (CLA), with antibody concentrations expressed as arbitrary units/mL relative to a positive control serum [31] (n = 277 samples); (ii) PCR on bone marrow biopsies using primers specific for kinetoplast DNA (kDNA) and ribosomal RNA [36] (n = 277 samples); (iii) rK39 Kalazar Detect Rapid Diagnostic Test (RDT), Inbios International Inc., WA., USA [37], (iv) qPCR primers for kDNA (described below), and (v) clinical score, defined as the sum of the score of six typical clinical signs (alopecia, dermatitis, chancres, conjunctivitis, onychogryphosis, and lymphadenopathy), each scored on a semi-quantitative scale from 0 (absent) to 3 (intense) [36] (n = 266 samples). Animals were assessed for infectiousness to the sandfly vector by xenodiagnosis, using uninfected colony-reared *Lutzomyia longipalpis*, and following dissection 4–5 days post full engorgement [22], [35]. Here, matching xenodiagnosis data were available for 103 dog bone marrow samples (36 infected dogs, 3,751 fed flies dissected), 58 dogs ear samples (26 infected dogs, 1,702 flies), 39 fox bone marrow samples (22 infected individuals, 1,309 flies), and 30 fox ear samples (18 foxes, 1,187 flies).

### Quantitative PCR (qPCR)

DNA was extracted from 100 µL aliquots of bone marrow, using phenol-chloroform [38]. DNA from 3 mm ear skin punch biopsies (average: 0.029 grams, range: 0.0144–0.0837) was extracted using a commercial kit (DNeasy: Qiagen, UK). qPCR was performed using primers specific for a conserved region of *Leishmania* kDNA [27]. Quantification of *Leishmania* DNA was performed by comparison of C_t_ values with those from a standard curve constructed from 10-fold dilutions of *L. infantum* DNA extracted from cultured parasites, from 1×10^5^ to 0.001 parasite equivalents/mL (strain MHOM/MA/67/ITMAP-263). Samples were tested in duplicate and standards in triplicate on every plate. The occasional duplicates giving one positive and one negative result were re-tested: none remained unresolved after re-testing. A non-template control (NTC) was run in triplicate on every plate. A plate of negative controls including DNA extracted from blood samples of 30 UK dogs with no history of foreign travel, and 40 endemic control dogs from São Paulo, Brazil was run every 5 plates. A standardised C_t_ threshold value of 0.01 was selected as cut-off value to define infection based on the NTC signal. The endogenous control was a eukaryotic 18S rRNA gene as a reference of total canine DNA quantified in a separate qPCR reaction to the *Leishmania* assay using pre-developed TaqMan Assay reagents (Applied Biosystems, UK) following the manufacturer's recommendations. Parasite loads were normalized (*d*) between animals to the eukaryotic 18S rRNA gene per reaction, where *d* = absolute *Leishmania* kDNA equivalents/(copy number of 18S rRNA gene/2)/ng tissue DNA extracted measured spectrophotometrically. Normalized log_10_ parasite numbers and absolute log_10_ parasite numbers per ml (bone marrow) or per gram (ear skin) were strongly correlated (r^2^ = 0.93 and r^2^ = 0.98 respectively). Consequently, for ease of interpretation, we report the per unit absolute log_10_ parasite numbers.

### Definition of infection and infectiousness

The date of patent infection for dogs and foxes was estimated as the first date at which animals were positive by any serological or parasitological assay; all samples thereafter were considered as infected based on previous analyses demonstrating a very low incidence of serological reversal [31], [35], [36]. At each bimonthly examination, dogs were classified according to their total clinical score as asymptomatic (scores 0–2), oligosymptomatic (3–6) and symptomatic (>6). Dogs with >8 months post infection follow-up and all bimonthly clinical scores <3 were considered long-term asymptomatic. Infectiousness was assessed as either positive (≥1 sandfly infected) or negative, or as the proportion of sandflies infected at any single time point (point xenodiagnosis). Dogs were also classified previously [22], [35] as “highly infectious” (>20% of total flies infected), “mildly infectious” (>0% and <20% flies infected), and “non-infectious” (no flies infected) by serial xenodiagnoses (n = 6,002 flies dissected from 173 independent trials): the highly infectious group were shown to be responsible for >80% of all transmission events [22]. All foxes were non-infectious (n = 1,469 flies from 44 trials) [35].

### Data analysis

Parasite aggregation was characterised by the dispersion coefficient *k* of the fitted negative binomial distribution. Negative binomial models were used to test for differences in parasite loads between groups. Analysis of parasite loads against independent variables were conducted using negative binomial mixed models, with animal identity included as the random effect. The relationship between infectiousness and markers of infection was analysed by logistic regression.

Receiver Operating Curves (ROC) were used to identify parasite load (qPCR) and anti-*Leishmania* antibody (ELISA) threshold values that maximised test sensitivity and specificity to differentiate currently infectiousness and non-infectious dogs. Areas under the ROCs were similar: 0.937 (ear biopsies, n = 58), 0.837 (bone marrows, n = 103) and 0.846 (ELISA, n = 173) (χ^2^ = 72.0, df = 2, *P* = 0.699, n = 52), providing test threshold values of 4.64 log_10_ parasites/gram (ear biopsies), 3.51 log_10_ parasites/mL (bone marrows), and 4.59 log_10_ antibody units/mL, respectively. These values were then used to evaluate the performance of threshold-based qPCR and ELISA assays to detect dogs classified by longitudinal infectious status in the mixed population. The average times of detection by the threshold-based assays relative to infection were calculated using Kaplan-Meier survival analysis. Differences in Kaplan-Meier curves were compared by log rank test, and confidence limits calculated following [39]. All analyses were carried out in Stata v.11.1 (Stata Corporation, College Station, Texas, USA).

## Results

### *Leishmania* loads of infected dogs

**Figure 1:**
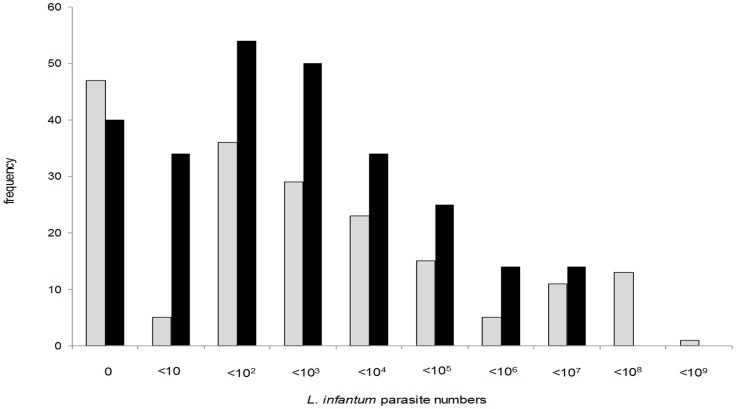
Frequency distributions of *L. infantum* parasite numbers in dog tissue samples measured by qPCR. Parasite numbers are expressed as numbers per mL (bone marrow, black bars) or per gram (ear skin, light bars).

Parasite loads were quantified by qPCR in 265 post-infection bone marrow samples from 82 dogs, and 185 post-infection ear skin biopsies from 64 dogs (Table 1). The median parasite loads were 142 parasites/mL in bone marrow and 119 parasites/gram in ear skin (Table 1) but the correlation was not strong (Spearman's ρ = 0.56, P<0.001). Note that since the unit of measurement of these two samples differ, the magnitude of the parasite loads in skin and bone marrow were not directly compared. The frequency distributions of parasite loads in both tissues was highly skewed, with maximum burdens of 2.4×10^6^ parasites/mL and 1.3×10^8^ parasites/gram in bone marrow and ear skin, respectively (Figure 1). The degree of parasite aggregation, measured by the negative binomial parameter *k*, was very high, with loads in ear skin (*k* = 0.066) showing greater aggregation than those in bone marrow (*k* = 0.104). Comparable degree of aggregation was observed for mean parasite loads in individual dogs (Table 1). Of the total *L. infantum* loads recorded in bone marrows biopsies, 90% of parasites were found in 8% (21/265) of samples and 16% (13/82) of dogs; for skin biopsies, the equivalent figures were 8% (14/185) of samples and 9% (6/64) of dogs.

**Table 1:** Comparison of the *L. infantum* parasite loads, and their degree of aggregation, in sympatric dog and fox populations in Amazon Brazil.

	n	mean	median	IQR	range	*k* (95% CL)
samples from infected dogs						
BM	265	1.0×10^5^	142	4–4194	0–2.4×10^6^	0.104 (0.091–0.120)
skin	185	2.7×10^6^	119	0–8670	0–1.3×10^8^	0.066 (0.056–0.079)
means for each infected dog						
BM	82	1.3×10^5^	1712	18–23835	0–2.2×10^6^	0.138 (0.108–0.175)
skin	64	3.9×10^6^	412	16–17936	0–1.3×10^8^	0.077 (0.058–0.102)
samples from infected foxes						
BM	67	2.9×10^4^	0	0–1744	0–1.1×10^6^	0.042 (0.029–0.062)
skin	53	8.4×10^4^	0	0–349	0–1.8×10^6^	0.047 (0.031–0.070)
means for each infected fox						
BM	34	2.5×10^4^	12	0–2167	0–5.3×10^5^	0.057 (0.034–0.094)
skin	30	6.3×10^4^	41	0–2893	0–8.3×10^5^	0.076 (0.048–0.121)

Statistics shown for individual samples and for means of all samples from each animal. Parasite values are expressed as number/mL (BM bone marrow) and number/gram (ear skin). IQR interquartile range; *k* negative binomial over-dispersion statistic (CL confidence limits).

**Figure 2:**
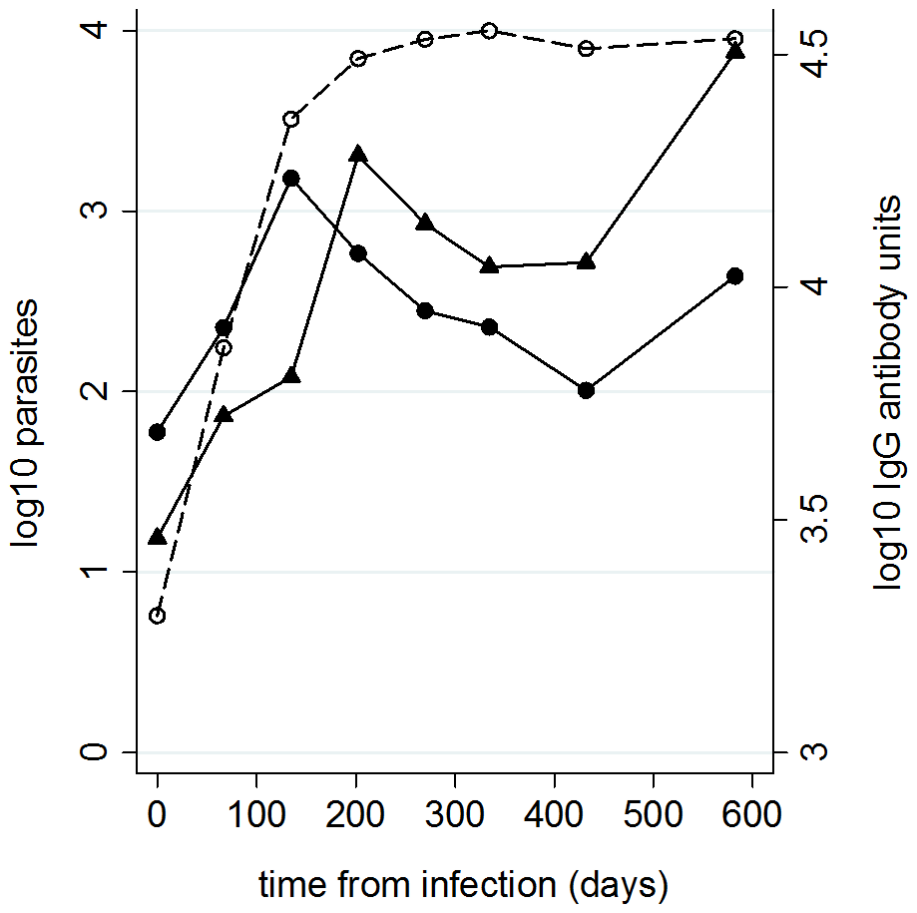
Average *L. infantum* parasite loads in dog tissues with time from infection. Parasite loads shown as log_10_ values for bone marrow (solid line, circles) and ear skin (solid line, triangles) biopsies. Anti-*Leishmania* IgG antibody units (dotted line, open circles) shown for comparison.

**Figure 3:**
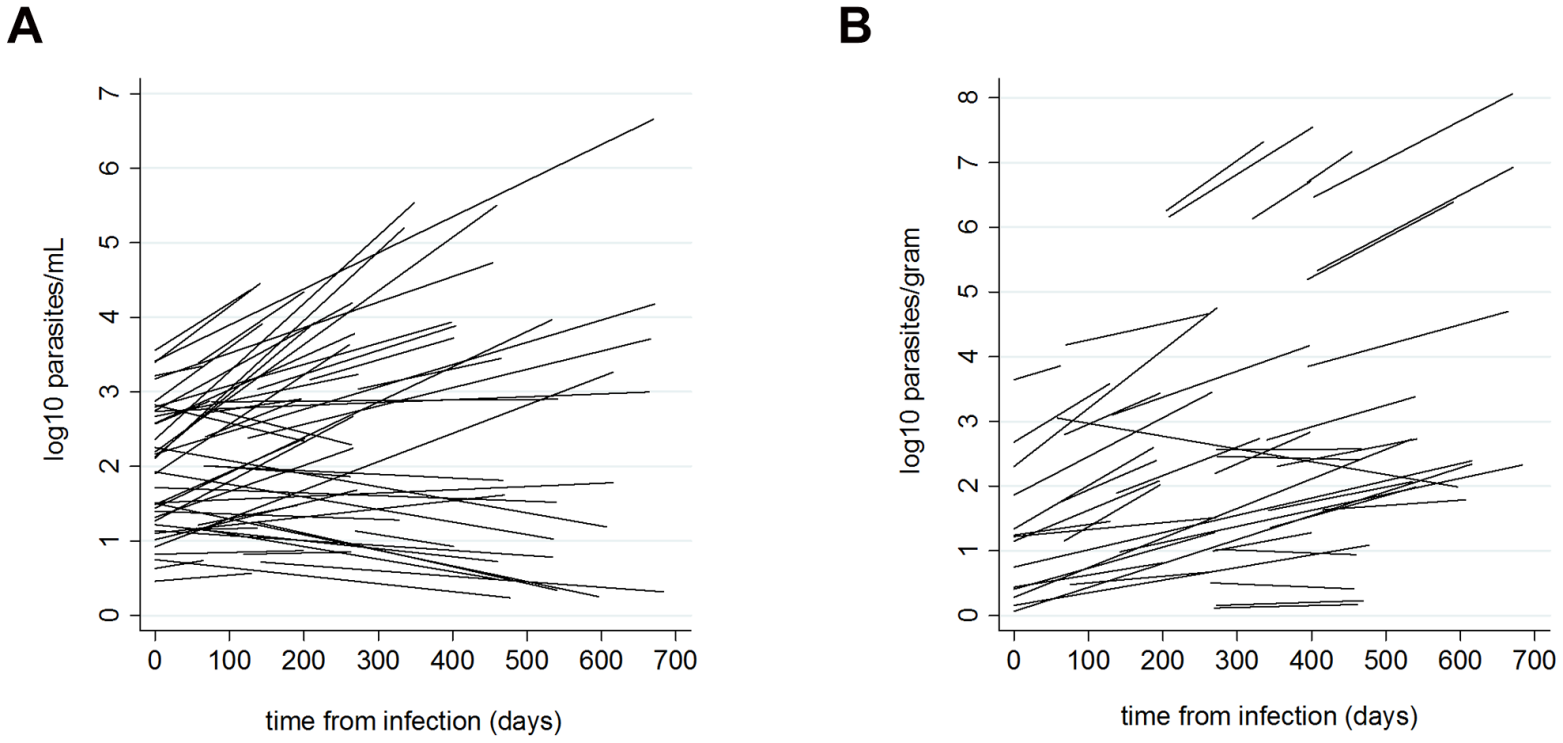
*L. infantum* parasite load model fits for individual dogs with time from infection. Lines represent fitted slopes of log_10_ parasite loads in (A) bone marrow (per mL) and (B) ear skin (per gram) examined using a multilevel mixed-effects time series model with random intercepts (initial loads) and slopes (parasite loads with time).

Parasite loads in both tissues increased on average with time since infection (Table 2; Figure 2). Ear skin loads increased at a faster average rate than bone marrow loads, reflected in the ear skin to bone marrow parasite load ratios being significantly greater in later infection (Table 2). However, the relationship between parasite load and time varied between individual dogs, showing positive to negative slopes for both tissues (Figure 3). Both bone marrow and ear skin loads were significantly higher in sick dogs, in infectious dogs and in dogs with higher anti-*Leishmania* antibody levels (Table 2). Severity of infection was also associated with greater ear skin to bone marrow parasite ratios (Table 2). However, in symptomatic dogs this ratio did not vary according to the type of symptom: dogs with skin symptoms had comparable ratios to those with only non-skin symptoms (IRR = 0.67 (95% CL 0.28–1.62), χ^2^ = 0.79, P = 0.37).

**Table 2:** Relationship between dog tissue *L. infantum* loads, time since infection, anti-*Leishmania* IgG, clinical status, and infectiousness.

Variable	Bone marrow	Ear skin	Ear skin/bone ratio^1^
Time infection (days)	1.001 (1.000–1.002) N = 265 Wald χ^2^ = 7.95, df = 1 P = 0.0048	1.003 (1.002–1.004) N = 185 Wald χ^2^ = 27.3, df = 1 P<0.0001	1.003 (1.002–1.004) N = 173 Wald χ^2^ = 26.6, df = 1 P<0.0001
Anti-*Leishmania* IgG (log units/mL)	1.54 (1.31–1.82) N = 265 Wald χ^2^ = 26.5, df = 1 P<0.0001	2.55 (1.96–3.32) N = 185 Wald χ^2^ = 48.7, df = 1 P<0.0001	2.11 (1.59–2.81) N = 173 Wald χ^2^ = 26.6, df = 1 P<0.0001
Infectious by xenodiagnosis^2^	2.20 (1.25–3.88) N = 58 Wald χ^2^ = 7.39, df = 1 P = 0.0066	4.27 (1.75–10.4) N = 58 Wald χ^2^ = 10.2, df = 1 P = 0.0014	1.69 (0.57–4.98) N = 52 Wald χ^2^ = 0.91, df = 1 P = 0.34
**Clinical status** 3			
Asymptomatic	1	1	1
Oligosymptomatic	1.26 (0.88–1.81)	2.06 (1.36–3.12)	2.11 (1.39–3.20)
Symptomatic	2.04 (1.36–3.05)	2.05 (1.25–3.37)	1.47 (0.89–2.41)
	N = 254 Wald χ^2^ = 11.8, df = 2 P = 0.0027	N = 185 Wald χ^2^ = 14.2, df = 2 P = 0.0008	N = 173 Wald χ^2^ = 12.3, df = 2 P = 0.0021

Coefficients expressed as incidence rate ratios. Data fitted to random effects negative binomial regression models, with time since infection as a covariate.

^1^Model includes ln bone marrow parasite number as a covariate.

^2^Infectiousness to sandflies measured by xenodiagnosis.

^3^Dogs were classified according to their total clinical score as asymptomatic (scores 0–2), oligosymptomatic (3–6) and symptomatic (>6) at each bimonthly examination.

### *Leishmania* loads and infectiousness to sandflies

**Figure 4:**
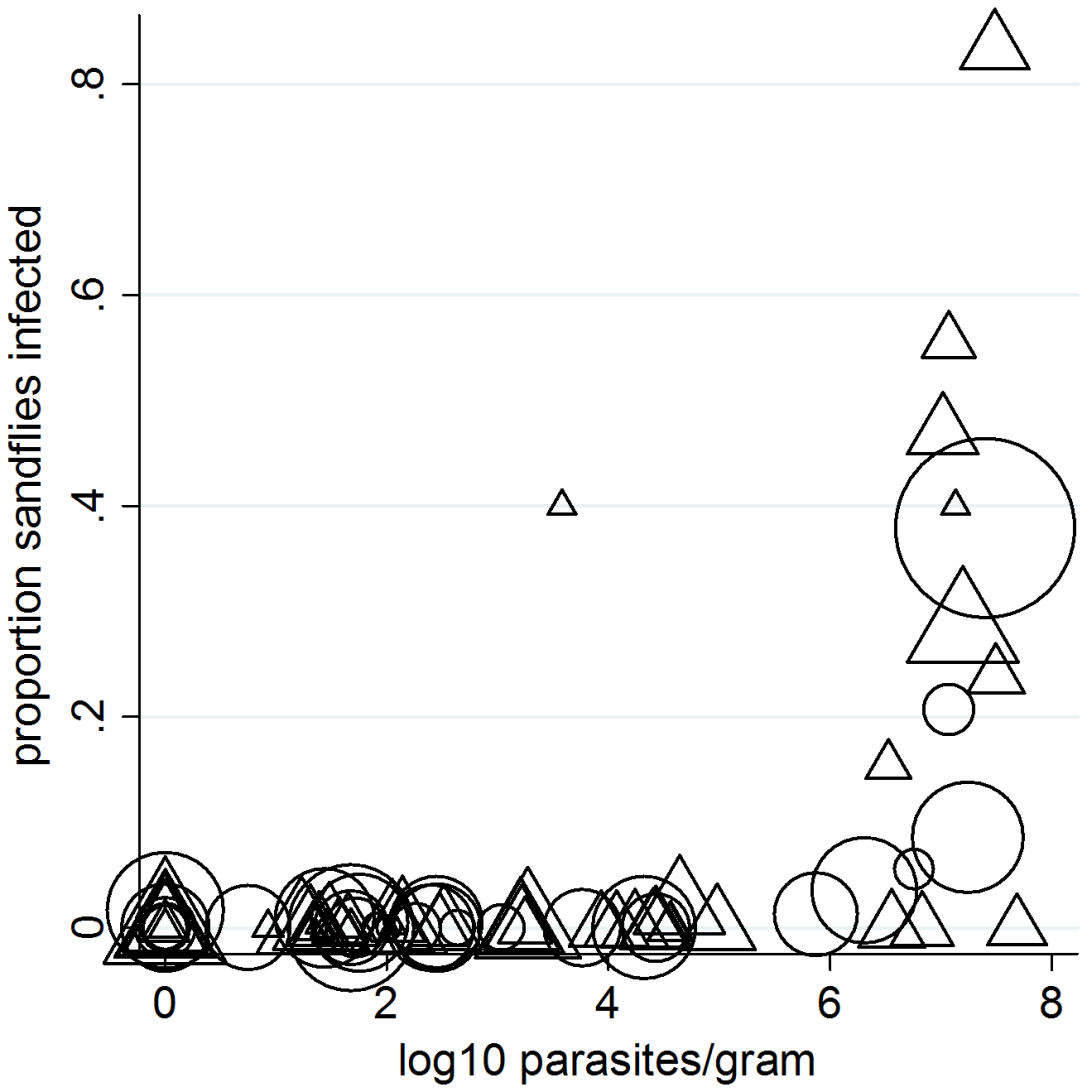
Relationship between *L. infantum* parasite loads in infected dog skin biopsies and infectiousness to sandflies. Individual dog infectiousness was measured as the proportion of exposed and blood-fed colony-reared *Lu. longipalpis* sandflies infected during xenodiagnosis. The figure shows results for individual samples (n = 58, triangles), and for the mean parasite load and proportion infected for each dog (n = 26, circles). Symbol size corresponds to sampling weight (number of fully engorged sandflies dissected).

**Figure 5:**
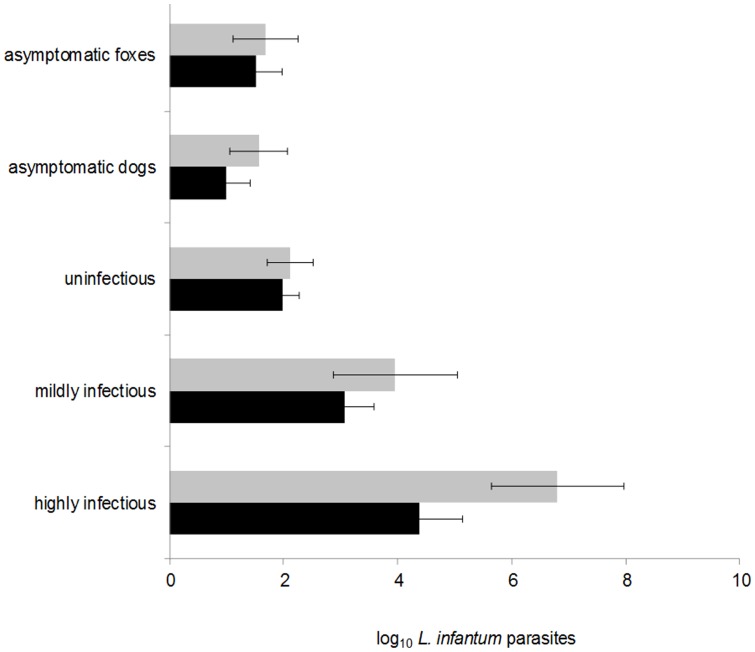
Average *L. infantum* parasite loads in dog and fox tissues. Parasite loads in bone marrow (light bars) and ear skin (dark bars) biopsy samples measured by real-time qPCR and expressed as mean (and 95% C.I.s) log_10_ parasites per mL (bone marrow) or per gram (ear skin). Samples from infected dogs are stratified by infectious status. Clinically asymptomatic dogs and foxes presented total clinical scores <3 at all clinical examinations over ≥8 months post infection follow-up. Infectiousness categories: highly infectious (n = 24 bone marrow samples; n = 9 ear skin samples); mildly infectious (n = 44, 26); uninfectious (n = 85, 60); asymptomatic dogs (n = 37, 27). Foxes were all asymptomatic (n = 67, 51).

The probability of a dog being infectious to sandflies at point xenodiagnosis was positively associated with parasite load, PCR status, IgG antibody titer, total clinical score, and time since infection; the strongest predictor of being infectious was ear skin parasite load (Table 3); similar results were seen when analysis was restricted to only paired bone marrow and ear skin samples (data not shown). Infectivity to sandflies was associated with high parasite loads in ear skin (Figure 4): the majority of dogs had loads <10^6^ parasites per gram and were very rarely infectious. Highly infectious dogs had higher mean parasite loads than mildly infectious dogs (ears: Wald χ^2^ = 7.36, P = 0.0073; marrow: χ^2^ = 7.21, P = 0.0067), the latter showing greater average loads than non-infectious dogs (ears: χ^2^ = 13.35, P = 0.0003; marrows: χ^2^ = 14.56, P = 0.0001) (Figure 5).

**Table 3:** Relationships between the probability of dogs being infectious to sandflies and immunologic, parasitological or clinical parameters.

Variable	n	OR	P	r^2^	AUC
Time since infection (days)	103	1.003	0.0369	0.044	0.649
Anti-*Leishmania* antibody^1^	103	5.685	0.0001	0.296	0.857
Total clinical score	93	1.285	0.0003	0.138	0.729
Dermatitis score	93	1.492	0.0006	0.127	0.708
Other clinical score	93	1.376	0.0065	0.079	0.672
PCR	103	26.1	0.002	0.248	0.712
Bone marrow load^1^	103	2.224	0.0001	0.258	0.837
Ear skin load^1^	58	2.78	0.0001	0.488	0.937

The probability of being infectious to sandflies measured by xenodiagnosis of infected dogs. OR odds ratio; r^2^ logistic regression pseudo; AUC area under receiver operator curve; dermatitis score: score of clinical dermatitis in skin only; PCR: negative or positive by standard PCR;

^1^log_10_ transformed anti-*Leishmania* antibody units (per mL) measured by ELISA, and *L. infantum* numbers in bone marrow (per mL), and ear skin biopsies (per gram), measured by qPCR.

### *Leishmania* loads in crab-eating foxes

*L. infantum* was detected in bone marrow of 50% (17/34) and skin of 67% (20/30) of infected foxes. Parasite loads showed similar over-dispersion as for dogs (Table 1). Of the total *L. infantum* loads recorded in bone marrows, 90% was attributed to 8% (5/67) of samples and to 12% (4/34) of the foxes. The equivalent figures for skin biopsies were 8% (4/53) of samples and 10% (3/30) of foxes.

Bone marrow loads varied significantly with fox age, rising rapidly in the first 6 months of life (age (months): IRR = 1.25 (95% CL 1.11–1.42), P = 0.0004) and declining thereafter (months^2^: IRR = 0.997 (0.995–0.999), P = 0.0015); a similar, though not significant, pattern was seen for ear skin samples (P = 0.25) (Supplementary Figure S1). No parasites were detected in 15 bone marrow samples from 6 foxes over 6 years old, whereas 4/6 of these foxes (4/12 samples) showed residual parasites in ear skin. In contrast, anti-*Leishmania* IgG titres did not decline in older age classes (Supplementary Figure S1). There were significant positive relationships between fox tissue parasite numbers and anti-*Leishmania* IgG titres (marrow Wald χ^2^ = 16.0, df = 1, P = 0.0001; skin Wald χ^2^ = 5.68, df = 1, P = 0.017), and ear skin to bone marrow parasite ratios were moderately higher in foxes with high titres (Wald χ^2^ = 3.81, df = 1, P = 0.051). Only one fox showed any clinical signs of disease (alopecia) but which was mild and transitory.

### Comparison of dogs and crab-eating foxes

Skin and bone marrow parasite loads of foxes were similar to those in non-infectious dogs (P>0.10) (Figure 5). Seven long-term “truly” asymptomatic infected dogs were identified: they transmitted infection to 1/678 sandflies exposed in 24 xenodiagnosis trials on 4 dogs. Their parasite loads were similar to those in foxes (P>0.18), which were all asymptomatic by the same definition (Figure 5). None of the 22 infected foxes tested were infectious in 39 xenodiagnosis trials. Applying the model coefficients from analysis of dog infectivity (Table 2) to fox ear skin parasite data (n = 53), foxes were predicted to have been infectious with ≥15% probability (≥10^4.64^ parasites/gram in skin) on 6 of 53 occasions for 4 foxes, equivalent to a total predicted number of infectious samples of 2.9 of 53, compared to the observed 0/39 xenopositive trials of infected foxes.

### Detecting infectious dogs based on *Leishmania* loads

The performances of qPCR and ELISA to differentiate dogs of different infectious status in the mixed population were tested using positivity threshold values calculated by ROC analysis of the point xenodiagnosis data (see Methods). PCR-based diagnostic tests showed a high sensitivity (94–100%) to detect highly infectious dogs, though the sensitivities of serology-based tests were somewhat lower (78–100%) (Table 4). The sensitivities of most tests to detect mildly infectious dogs were lower, but these dogs contributed <20% of transmission. Only tests based on qPCR thresholds showed high specificities for infectious dogs (i.e. low sensitivities to detect non-infectious dogs) (Table 4). Highly infectious dogs were detected by qPCR significantly earlier after patent infection (152 days [95% CI: 117–186]) than either mildly infectious dogs (442 days [302–582]) or non-infectious dogs (435 days [317–553]) (log rank tests: qPCR: χ^2^>17.3, P<0.0003); estimates for the latter two groups were statistically indistinguishable (P = 0.70). Detection time of highly infectious dogs approximated their observed time to becoming infectious (134 days [68–201]).

**Table 4:** Sensitivity and specificity of diagnostic tests to detect dogs of different infectious status.

	Proportion of samples positive (n/total)		
	Highly infectious^1^	Mildly infectious	Non-infectious
*Test thresholds to detect infectivity* 2			
Ear cut-off	1.00 (8/8)	0.50 (14/28)	0.02 (1/64)
BM cut-off	0.94 (17/18)	0.35 (16/46)	0.19 (15/79)
ELISA cut-off	0.82 (27/33)	0.38 (29/76)	0.32 (44/137)
*Conventional tests to detect infection* 3			
Ear positive	1.00 (8/8)	1.00 (28/28)	0.78 (50/64)
BM positive	1.00 (18/18)	0.83 (38/46)	0.81 (64/79)
ELISA positive	0.97 (32/33)	0.83 (63/76)	0.87 (120/138)
PCR positive	1.00 (24/24)	0.58 (32/55)	0.48 (40/84)
rK39 positive	0.79 (22/28)	0.57 (34/60)	0.50 (49/98)

^1^Dogs were classified as highly infectious, infectious, and non-infectious to the sandfly vector *Lu. longipalpis* by longitudinal xenodiagnosis follow-up.

^2^Test performance based on parasite numbers, or IgG antibody units, calculated at point xenodiagnosis to detect infectiousness of infected dogs.

^3^Test performance based on conventional cut-offs to detect infection.

## Discussion

We demonstrate pronounced heterogeneity in *L. infantum* loads between dogs, assessed by qPCR in bone marrow and ear skin. Loads were highly over-dispersed with evidence of greater aggregation in ear skin relative to bone marrow (9% *vs* 16% of dogs harboured 90% of total parasites). Parasite loads in the two tissues showed different dynamics: bone marrow loads increased rapidly reaching a peak 100–200 days after infection, while ear skin loads continued to increase over a 600 day period, resulting in increased skin to bone marrow load ratios in late infection. Dissemination to the skin varied between dogs, being greater in sick and infectious dogs. Evidence of *L. infantum* parasite over-dispersion has been reported in different dog tissues [27], [40]–[42] and in human blood [15], [26], [43], and greater variation in parasite loads in ear skin compared to paired bone marrows, lymph nodes, blood, and liver and spleen samples has been reported for Brazilian dogs [40], [42]. However these studies did not evaluate parasite loads through time. One cohort study of Italian dogs noted a decrease in ear to lymph node parasite ratios during clinical development, in apparent contrast to results here. In that study, the time of infection was not established, so dogs may have been at a different stage and severity of infection [34].

Tissue parasite load, particularly in ear skin, was the best predictor of being currently infectious to vectors. *L. infantum* amastigotes in skin tissue or skin capillaries are directly accessible to sandflies, which are known to feed abundantly on ear pinnae; and ear skin appears to be more infective than abdomen skin [23], [30]. Some of the variation in parasite loads between ear tissue samples may also reflect small scale spatial variation in parasite density within the ear. We did not restrict sandflies to feed only on ears, unlike other studies [23], [29], [44]. However, the proportion of dogs that were infectious was substantially lower than the proportion with detectable skin parasites, and only dogs with very high skin parasite loads were consistently infectious. Highly infectious dogs showed greater average loads compared to mildly infectious and non-infectious dogs, and also tended to fall within the top 20% parasite loads for each tissue. These data, and the observed high degree of parasite aggregation in ear skin, suggest that the majority of transmission events to vectors result from a small proportion of infectious dogs. Previously we reported for these dogs that 7 of 42 infectious dogs (17%) were responsible for >80% of all sandfly infections [22]. Similar over-dispersion in infectiousness can be calculated from published xenodiagnosis studies, with 15% to 44% of dogs accounting for >80% of transmission events [23], [44]. qPCR studies of canid tissue *L. infantum* loads relative to xenodiagnoses are not available elsewhere, but parasite estimates by immunohistochemistry of ear skin show moderate correlations with xenodiagnosis positivity [30], [45]. Our current results suggest that high parasite loads in dog ear skin, rather than the simple presence of parasites, is the important metric to identify likely infectious individuals and potential reservoir populations. In the current study, all infections were shown to be *L. infantum* [36]. To identify super-spreaders in regions of mixed *Leishmania* co-infections, the specificity of qPCR methods would need to be fully validated.

Current ZVL control strategy in Brazil includes mass test-and-slaughter of *Leishmania* antibody positive dogs [17], which is criticised on theoretical, logistical and also on ethical grounds [18]–[22]. If the small fraction of dogs that are responsible for the majority of transmission could be identified (e.g. by detection of high parasite loads) and targeted, this would directly address many of these issues, and may be more cost-effective than mass interventions [9], [12]. Canine infectiousness to sandflies is known to increase with the severity of disease and high anti-parasite antibody, but sensitive and specific markers of infectiousness have not been identified [22], [23], [29], [30], [46]. Here, we show that adopting quantitative test threshold values based on skin parasite numbers, highly infectious dogs can be distinguished from non-infectious dogs. These tests were highly sensitive for highly infectious dogs, equivalent to detection of 87–94% of sandfly infections in these samples (data not shown), and importantly also showed high specificities (0.83–0.99) to detect non-infectious dogs, unlike conventional tests for infection. Since up to 50% of seropositive dogs may be asymptomatic in a single community survey, such a targeted approach should also raise dog-owner compliance.

The crab-eating fox occurs widely in South America, and is commonly infected with *L. infantum* [16], [35], [47], and thus often assumed to be a sylvatic reservoir. However, few infected foxes have been shown to infect sandflies [48], [49], and in our cohort study none of the foxes were infectious [35]. Here, we show that fox parasite loads, though heterogeneous, were significantly lower than those of infectious dogs, and similar to non-infectious dogs, providing further evidence that foxes are not likely to be important for maintaining transmission [22], [35]. The results also provide a parasitological explanation for why the foxes here, and probably wild canids more generally, tend to present asymptomatic infections [16], [50], [51]. Relatively low parasite loads were also noted in the truly asymptomatic cohort dogs, as also reported in asymptomatic human infection [26], [28], [52]. Whether asymptomatic human infections with *L. donovani* is associated with low parasite loads and thus low transmission potential remains speculative, and further studies are needed [16]. Variation in parasite load between individuals of other potential reservoir hosts (e.g. hares in Iberia [53]), and variation in parasite load in skin between different parts of the host, would also be informative.

In conclusion, this study highlights the importance of quantifying heterogeneities in infection loads in relation to transmission potential through prospective studies, underpinning development of novel tools for parasitic disease management. Studies are now needed to confirm the efficacy of diagnostic threshold-based driven actions against transmission, and to develop diagnostic kits, based on the detection of parasite DNA (e.g. isothermal amplification) or parasite antigens, for practical field use.

## Supporting Information

Figure S1 **Average** ***L. infantum*** **parasite loads in fox tissues with increasing fox age.** Average log_10_ *L. infantum* parasite loads in ear skin biopsies (per gram) (solid line, triangles) and bone marrow aspirates (per mL) (solid line, circles) with fox age-class in a naturally infected crab-eating fox population. Also shown are log_10_ anti-*Leishmania* IgG antibody units (per mL) (dotted line, open circles) for comparison. Data are shown for infected foxes only.
(TIF)
