# Supplementary figures and images for: Heterogeneities in Leishmania infantum Infection: Using Skin Parasite Burdens to Identify Highly Infectious Dogs
Source: PLoS Negl Trop Dis. 2014 Jan 9;8(1):e2583. doi: 10.1371/journal.pntd.0002583 (PMC3886905; doi:10.1371/journal.pntd.0002583)

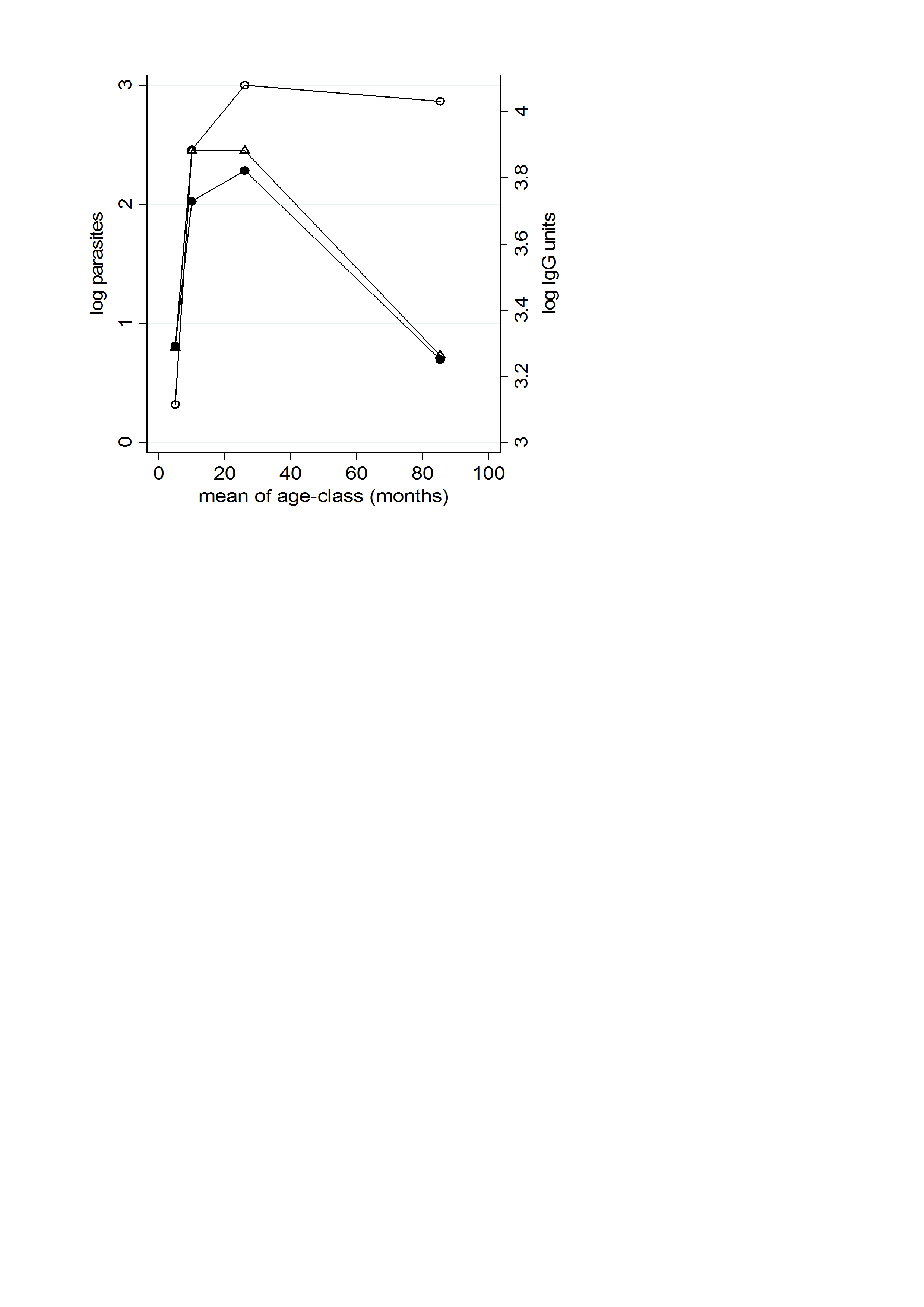

Supplement: Figure S1 — Average L. infantum parasite loads in fox tissues with increasing fox age. Average log10 L. infantum parasite loads in ear skin biopsies (per gram) (solid line, triangles) and bone marrow aspirates (per mL) (solid line, circles) with fox age-class in a naturally infected crab-eating fox population. Also shown are log10 anti-Leishmania IgG antibody units (per mL) (dotted line, open circles) for comparison. Data are shown for infected foxes only. (TIF) [file pntd.0002583.s001.tif]
